# The Epidemiology of Post-Traumatic Stress Disorder and Alcohol Use Disorder

**DOI:** 10.35946/arcr.v39.2.02

**Published:** 2018

**Authors:** Nathan D. L. Smith, Linda B. Cottler

**Affiliations:** Nathan D. L. Smith, A.L.M., is a doctoral student in the Department of Epidemiology, University of Florida, Gainesville, Florida. Linda B. Cottler, Ph.D., M.P.H., F.A.C.E., is the dean’s professor in the Department of Epidemiology, University of Florida, Gainesville, Florida

**Keywords:** alcohol use disorder, epidemiology, NESARC, post-traumatic stress disorder, veterans

## Abstract

For more than 40 years, research has shown that individuals with post-traumatic stress disorder (PTSD) use alcohol and experience alcohol use disorder (AUD) to a greater degree than those with no PTSD. AUD and PTSD have shown a durable comorbidity that has extended through decades and through changes in disorder definitions. Some research shows that veterans who have experienced PTSD have a high likelihood of developing AUD, perhaps reflecting the self-medication hypothesis. Other research shows that people with substance use disorder are likely to be exposed to traumatic situations and develop PTSD. These two areas of research could represent two separate relationships between PTSD and AUD. Finally, there is still no clear determination of which cluster of PTSD symptoms is most closely associated with AUD.

## Introduction

The harmful use of alcohol has been of interest to doctors for centuries, and minimizing the harm caused by alcohol use disorder (AUD) has been a priority of psychiatrists in the United States since at least 1917.^1^ However, although traumatic experiences are ubiquitous throughout human history, it was only after the Vietnam War that psychiatrists codified the harms caused by traumatic stress into a distinct diagnosis.^2^ For more than 40 years, it has been known that individuals with post-traumatic stress disorder (PTSD) use alcohol and experience AUD more than those with no PTSD. This link between PTSD and AUD subsequently has been broadened beyond Vietnam veterans to include veterans of other wars and anyone exposed to trauma. The considerable psychological distress caused by AUD and PTSD, both separately and together, affects the lives of millions of men and women, including underrepresented populations, such as people with other mental health conditions.

## Disorder Definitions

This section provides an overview of commonly used definitions and how they have changed over time.

### AUD

In 1952, the first edition of the *Diagnostic and Statistical Manual of Mental Disorders* (DSM) included “alcoholism” as one of two disorders under the category of “addiction.”^3^ The pithy, two-sentence definition instructed that an alcoholism diagnosis be used in cases of “well-established addiction to alcohol.” Since then, the definition of what is now called AUD has been significantly expanded and refined for each edition of the DSM.^2^,^4^–^7^

The third edition of the DSM (DSM-III) was published in 1980. In this edition, the disorders were called “alcohol abuse” and “alcohol dependence.”^2^ A diagnosis of alcohol abuse required:

- A “pattern of pathological alcohol use,” which was defined by features such as the need for daily alcohol consumption to function, the inability to reduce or stop drinking, remaining intoxicated for at least 2 days, or blackouts
- “Impairment in social or occupational functioning due to alcohol use,” which could include violent behavior, absences from work, or losing a job
- “Duration of disturbance of at least 1 month”

A diagnosis of alcohol dependence required the first two criteria of alcohol abuse, along with indications of tolerance (the need to increase the amount of alcohol to achieve the desired effect) or withdrawal (the development of physical symptoms after reducing or discontinuing alcohol consumption).

The 1987 revision of the third edition, the DSM-III-R, introduced major diagnostic changes for alcohol-related disorders. In the DSM-III-R, an “alcohol dependence” diagnosis required three out of nine possible criteria, and an “alcohol abuse” diagnosis required only two.^5^ The diagnosis of alcohol abuse was to be used only for individuals who had alcohol-related problems but did not meet the requirements for alcohol dependence. The DSM-IV diagnoses were substantially similar to those in the DSM-III-R.^6^

In the DSM-5, the terms “alcohol dependence” and “alcohol abuse” were removed, and the two separate diagnoses were replaced with one diagnosis—AUD.^7^ The DSM-5 lists 11 symptoms for the disorder, and an AUD diagnosis now has levels of severity based on the number of symptoms presented. The presence of two to three symptoms indicates mild AUD, four to five symptoms indicate moderate AUD, and six or more symptoms indicate severe AUD.

### PTSD

Unlike AUD, PTSD has only been included in the DSM since the third edition. In one of the first published articles on the occurrence of PTSD in the general population, Helzer and colleagues described the inclusion of PTSD in the DSM-III as a “compromise” for veterans’ groups and mental health personnel advocating for recognition of what was commonly called “post-Vietnam syndrome.”^8^ Adding PTSD as a possible diagnosis for anyone who had experienced a trauma was a middle ground between those who hypothesized that the disorder was unique to Vietnam veterans and those who believed it might not exist at all.

In the DSM-III-R and DSM-IV, a PTSD diagnosis was defined by experiencing a qualifying traumatic event (Criterion A) and three other clusters of symptoms: re-experiencing the event (Criterion B), emotional numbing and avoidance of cues and reminders of the event (Criterion C), and hyperarousal (Criterion D).^5^,^6^ King and colleagues conducted a factor analysis on the Clinician-Administered PTSD Scale, a measurement tool based on the DSM-IV diagnostic criteria, and found that these four clusters of symptoms best defined the disorder.^9^ This four-cluster model subsequently has been used in many examinations of the connections between PTSD symptoms and alcohol use.

The definition of PTSD was updated significantly for the DSM-5.^7^ The major changes included:

- Reclassification of PTSD as a trauma- and stressor-related disorder instead of an anxiety disorder
- Elimination of the criterion that the person’s response to the traumatic event must involve intense fear, helplessness, or horror
- Addition of the requirement that the symptoms cannot be attributed to the physiological effects of substance misuse, a medication, or another medical condition

### Conditional disorders

Both PTSD and AUD are conditional disorders; that is, both disorders can be diagnosed only if certain prerequisite conditions are met—specifically, a traumatic event or alcohol use. In the DSM-III, the prerequisite condition for PTSD was “existence of a recognizable stressor that would evoke significant symptoms of distress in almost everyone.”^2^ In the same edition, the section on substance use disorder (SUD) referred to “the maladaptive behavior associated with more or less regular use of the substances.”

Importantly, analyses can be conducted on the risk for the exposure to an event among the entire population, and then among those who experienced an event. Social determinants of health for the diagnoses may vary considerably based on likelihood of being exposed to an event or exposure to a substance. Conversely, risk for who later develops a diagnosis, given exposure, may be different as well. For this reason, it is important to evaluate both risk for exposure as well as risk for a disorder among those exposed.

## Prevalence Surveys in the United States

Since the late 1970s, several U.S. surveys have collected information on mental health conditions, including AUD, SUD, and PTSD. These surveys include the Epidemiological Catchment Area (ECA) program, the National Comorbidity Survey (NCS), and the National Epidemiologic Survey on Alcohol and Related Conditions (NESARC).

### ECA

In 1978, the President’s Commission on Mental Health concluded that the existing body of research could not answer these fundamental questions: What is the prevalence of mental health conditions in the United States, and are people with mental health conditions receiving adequate treatment?^10^ The ECA was designed to answer these questions.^11^ Although the ECA study did not include a nationwide sample, sites were chosen to be representative of the U.S. population and included Baltimore, Maryland; Durham, North Carolina; Los Angeles, California; New Haven, Connecticut; and St. Louis, Missouri. The ECA program used the National Institute of Mental Health (NIMH) Diagnostic Interview Schedule (DIS) to conduct face-to-face interviews with more than 20,000 people.^12^,^13^ The NIMH DIS questions were based on DSM-III diagnostic criteria. At all five sites, information on alcohol use was collected, and the St. Louis location also assessed traumatic event experiences and PTSD.^8^

The ECA program reported that the lifetime prevalence of DSM-III alcohol abuse and dependence was almost 14%.^14^ Prevalence varied by location, from about 11% in New Haven and Durham to about 16% in St. Louis. Individuals who had problems with alcohol were almost three times as likely to have a co-occurring mental disorder as those with no alcohol problem. Antisocial personality disorder and SUD were the most common co-occurring disorders.

The information collected at the St. Louis location provided one of the first estimates of the prevalence of PTSD in the general population. Of the 2,493 participants, about 16% were exposed to at least one qualifying traumatic event.^8^ Of this group, about 8.4% developed PTSD.^15^ Also, individuals who met criteria for PTSD were more likely to report alcohol-related problems than those who did not meet PTSD criteria.

### NCS

The Survey Research Center at the University of Michigan’s Institute for Social Research conducted a national study of comorbidity between 1990 and 1992.^16^ Trained interviewers administered a modified version of the World Health Organization’s Composite International Diagnostic Interview (CIDI), which was based on the DIS, to 8,098 individuals representing the contiguous 48 states. The NCS used the DSM-III-R definitions to assess alcohol dependence, alcohol abuse, and PTSD.

In the NCS sample, qualifying PTSD traumatic events were reported by 61% of men and 51% of women.^16^ Although more men reported experiencing traumatic events than women, women who experienced trauma were more than twice as likely than men to develop PTSD (20% vs. 8%). About 14% of the sample met criteria for lifetime alcohol dependence.^17^ Also, respondents who met criteria for PTSD were more than twice as likely to report co-occurring alcohol abuse or dependence, and they were almost three times as likely to report drug abuse or dependence.^16^

### NESARC Waves 1 and 2

The NESARC studies conducted in 2001 to 2002 (Wave 1) and 2004 to 2005 (Wave 2) collected nationally representative data on AUD and other mental disorders using the Alcohol Use Disorder and Associated Disabilities Interview Schedule (AUDADIS), which was designed by the National Institute on Alcohol Abuse and Alcoholism (NIAAA). The AUDADIS interview questions, heavily based on the CIDI, used DSM-IV criteria. NESARC Wave 2 consisted of 34,653 face-to-face interviews with individuals previously interviewed in Wave 1.^18^ According to data from Wave 2, the lifetime prevalence of alcohol abuse was found to be about 27% for men and 13% for women, and the lifetime prevalence of alcohol dependence was about 21% for men and 10% for women.^19^

The survey data showed that 77% of the respondents had experienced a qualifying traumatic event, as defined by the DSM-IV.^18^ The most commonly reported stressful life events were indirect experience of 9/11, serious illness or injury to someone close, and unexpected death of someone close. Of those who had experienced a trauma, about 8% developed PTSD. Individuals with PTSD were more likely to report mood disorders, anxiety disorders, SUD, and suicidal behavior than respondents without PTSD. Also, respondents with PTSD were more likely than those without PTSD to have co-occurring AUD, after controlling for sociodemographic factors such as age and race. However, this association was no longer significant when the analysis controlled for other co-occurring mental health conditions in addition to the sociodemographic characteristics.

### NESARC-III

The most recent NESARC interviews, conducted between 2012 and 2013, included a representative sample of 36,309 adults in the United States, and DSM-5 criteria were used.^20^ According to data from the NESARC-III, lifetime prevalence of AUD was 29%, and past 12-month prevalence was about 14%.^21^ Prevalences were higher among men, Whites, Native Americans, younger adults, and those who were previously married or never married. The lifetime prevalence of severe AUD was about 14%, and the past 12-month prevalence was more than 3%. Less than 20% of respondents who experienced AUD in their lifetime ever sought treatment for the condition.

In the NESARC-III sample, about 69% of respondents had experienced a qualifying traumatic event.^22^ Of this group, almost 9% met lifetime criteria for PTSD, and almost 7% met the criteria in the previous 12 months. Rates were higher among younger adults, Whites, Native Americans, and those with less education and lower incomes. PTSD was significantly associated with other psychiatric conditions, such as SUD, mood disorders, anxiety disorders, and personality disorders. Specifically, respondents who had PTSD, versus those who did not, were 1.5 times as likely to meet criteria for SUD and 1.2 times as likely to meet criteria for AUD in their lifetime, even after adjusting for other psychiatric disorders.

## Prevalence Surveys Outside the United States

Through many decades, despite numerous definition changes for each, AUD and PTSD consistently co-occur. This durable comorbidity has been found in large, small, representative, and targeted samples. U.S. surveys, such as the St. Louis sample of the ECA,^8^ the NCS,^16^ and the NESARC,^23^ have consistently found relationships between alcohol problems and PTSD.

Co-occurrence of AUD and PTSD has also been found in Europe, where rates of trauma exposure and PTSD vary greatly from country to country.^24^ In a 2004 analysis of a survey of the general population of six European countries, the European Study of the Epidemiology of Mental Disorders, which used the DSM-IV criteria for disorders, researchers reported that individuals with PTSD were twice as likely than those without PTSD to have co-occurring alcohol abuse and were three times as likely to have co-occurring alcohol dependence.^25^ An examination of the 1997 National Survey of Mental Health and Wellbeing, an Australian survey of more than 10,000 individuals, reported that about 1 in 4 individuals with PTSD also had AUD.^26^

## Co-Occurring Disorders

Some populations, such as military veterans and people with SUD, are at high risk for comorbidities, including co-occurring AUD and PTSD. For example, in one study of a sample of individuals seeking treatment for SUD, alcohol misuse was associated with meeting the criteria for a PTSD diagnosis.^27^ In another notable case, 141 Australian firefighters who had been exposed to a trauma and screened positively for potential PTSD were followed for several years.^28^,^29^ After 42 months, 42% of the participants had AUD, and 54% had experienced PTSD.

### PTSD before AUD

The consistent association between PTSD and AUD has led to debate about which condition develops first. One theory is that individuals with PTSD use alcohol and other substances to numb their symptoms and later develop AUD or SUD. This self-medication hypothesis was proposed by Khantzian to explain behavior exhibited by individuals with AUD and SUD who were being treated in a clinical setting.^30^ This theory has been supported by the demonstration of a mechanism that may encourage alcohol cravings. In laboratory settings, individuals with both AUD and PTSD reported increased cravings for alcohol after being presented with a trauma stimulus, as compared to a neutral stimulus.^31^ Other epidemiologic research has shown that a diagnosis of PTSD using the DSM-III-R criteria was predictive of later development of SUD.^32^,^33^ Trauma exposure alone, in the absence of a PTSD diagnosis, did not predict SUD.

Alternatively, some evidence shows that people exposed to trauma might be less likely to develop AUD after a traumatic experience. In a study of survivors of the Oklahoma City bombing in 1995, North and colleagues found that no new cases of AUD were reported after the bombing.^34^ This finding mirrors a previous study of individuals who experienced a mass shooting in 1991.^35^ In that study, three new cases of AUD were reported, but overall incidence of alcohol misuse significantly decreased in both men and women. These findings may indicate that some traumatic experiences bestow a type of survivor resilience that is protective against later development of AUD. Further research is needed to understand this phenomenon.

### AUD before PTSD

An alternative to the self-medication hypothesis was proposed in 1992. Using the St. Louis ECA, Cottler and colleagues hypothesized that individuals who had SUD may have been exposed to more circumstances that cause traumatic events.^15^ This heightened exposure may lead to experiencing more traumatic events and, ultimately, increase the likelihood of developing PTSD; although other explanations, such as AUD increasing sensitivity for developing PTSD, may also contribute. In the St. Louis ECA example, Cottler and colleagues confirmed their hypothesis, and they suggested that the use of substances such as opiates or cocaine led to even greater risk of exposure to traumatic events and an increased likelihood of developing PTSD.^15^

Several years later, this hypothesis was tested again in a sample of 464 drug users.^36^ In this study, the onset of drug use preceded exposure to traumatic events for men, but for women there was no difference in the timing of the events. A similar pattern of substance misuse leading to dangerous and traumatic experiences was found among African American women at risk for HIV.^37^ In a study that examined African Americans with SUD who were not receiving treatment, alcohol and substance misuse, with the exception of crack cocaine use, preceded the traumatic events.^38^ Finally, a longitudinal study of adults in Michigan found that PTSD predicted increased likelihood of SUD at a 5-year follow-up, but preexisting SUD did not predict later exposure to trauma or PTSD.^33^

### Prevalence in veterans

Drinking alcohol has been associated with the military for centuries. Military personnel use alcohol to cope with fear and other strong emotions experienced during and after combat.^39^ Combat is the traumatic event most strongly associated with PTSD, and the ECA found that about 20% of veterans who were wounded in the Vietnam War developed PTSD.^8^ More recently, veterans of the Iraq and Afghanistan wars who had PTSD were twice as likely to report alcohol misuse as those with no PTSD.^40^ More than 28% of veterans screened positive for alcohol misuse, and 37% screened positive for PTSD. Of those who met criteria for PTSD, 76% had co-occurring depression, which was more than twice the rate of depression among veterans who did not have PTSD. Similarly, a prospective study of service members in the United Kingdom found that those who had experienced combat increased their drinking more than those who had not been deployed.^41^ This finding was particularly strong for respondents who thought they might be killed or for those who experienced hostility from civilians while deployed.

Soldiers with PTSD who experienced at least one symptom of AUD may be disinhibited in a way that leads them to make risky decisions, including the potential for aggression or violence. One study conducted with veterans of the wars in Iraq and Afghanistan demonstrated a link between PTSD and AUD symptoms and nonphysical aggression.^42^ Veterans with milder PTSD symptoms who misused alcohol were more likely to perpetrate nonphysical aggression than veterans who did not misuse alcohol. However, this relationship was not demonstrated with significance among veterans who had more severe PTSD symptoms.

### Prevalence in women

Researchers continue to find more traumatic events and PTSD in women than in men. For example, in the NESARC Wave 2, lifetime prevalence of PTSD among women who experienced trauma was twice as high as the prevalence among similar men.^18^ A review of community samples reported that the prevalence of co-occurring SUD and PTSD among women is higher than the prevalence among men,^43^ and women who experienced abuse or neglect were significantly more likely to have AUD than controls.^44^ Higher prevalence in women compared to men has also been found in women who use illicit substances.^36^

Women who have experienced sexual assault or childhood sexual abuse appear to have particularly high rates of psychiatric disorders, including PTSD and AUD. In one notable study, women who self-reported childhood sexual abuse had an increased likelihood of having psychiatric disorders or SUD.^45^

## AUD and PTSD Symptom Clusters

Several studies have examined how the four clusters of PTSD symptoms (re-experiencing, effortful avoidance, emotional numbing, and hyperarousal) may affect how individuals develop and recover from PTSD and AUD. If some symptom clusters are closely associated with AUD, that information may be useful when screening people with PTSD for potential AUD. In an early study, hyperarousal symptoms were associated with AUD, whereas other clusters were not.^46^ However, later research found mixed results, with one study finding no relationship between any symptom cluster and AUD,^47^ and another study finding that the re-experiencing cluster was most strongly associated with alcohol problems.^48^ A study of veterans of the Iraq and Afghanistan wars found that the emotional numbing cluster, compared to the other symptom clusters, was significantly associated with alcohol misuse, even when controlling for other variables associated with AUD, such as depression and direct combat exposure.^40^ Finally, in a different study, a reduction of PTSD symptoms in each cluster was associated with less severe drinking overall, and a reduction in hyperarousal symptoms preceded positive changes in alcohol use.^49^

## Conclusion

The association between AUD and PTSD has been elucidated due to the development of standardized assessments for the ECA using the DSM-III DIS. Assessments that followed have used the foundational structure and question format of the DIS to interview participants. They include the CIDI, AUDADIS, and, recently, the Psychiatric Research Interview for Substance and Mental Disorders. In fact, the DIS has continued to be revised based on the DSM and the International Classification of Diseases, making it one of the most durable standardized diagnostic assessments in the field.

AUD and PTSD have shown a consistent comorbidity over many decades and in diverse populations. The strong relationship is present in representative surveys of the United States, throughout Europe, and in Australia. The relationship persists in studies of population subgroups at risk, such as veterans of the wars in Vietnam, Iraq, and Afghanistan; firefighters; women; and people with SUD. Although men have a higher prevalence of AUD than women, and women have a higher prevalence of PTSD than men, any individual with either disorder is more likely to have the other.

The evidence suggests that there is no distinct pattern of development for the two disorders. Some evidence shows that veterans who have experienced PTSD tend to develop AUD, perhaps reflecting the self-medication hypothesis. However, other research shows that people with AUD or SUD have an increased likelihood of being exposed to traumatic situations, and they have an increased likelihood of developing PTSD. It is possible that these two bodies of evidence represent two separate relationships between PTSD and AUD. Additionally, the conditional nature of the disorders, based on the exposure to an event or a substance, makes this a complex relationship for analysis, interpretation, and intervention for treatment.

Currently, there are several questions that remain unanswered. How different are the outcomes of the disorders when one or the other develops first? Are any of the PTSD symptom clusters more likely to lead to AUD? Are there particular traumatic experiences that provide some resilience against developing AUD? Are there significant differences in the occurrence and trajectory of PTSD and AUD among racial and ethnic minorities? These questions, and others, should be addressed by further research to ultimately minimize the harm experienced by the millions of individuals who experience AUD and PTSD.
